# *Psidium* Defenses Against *Meloidogyne enterolobii*: Proteomic and Microscopic Analysis of this Plant‐Predator Association

**DOI:** 10.1002/pmic.70015

**Published:** 2025-07-26

**Authors:** Sara Nállia de Oliveira Costa, Roberta Pena da Paschoa, Camilla Ribeiro Alexandrino, Pamela Maciel Cremonez, Juliana Martins Ribeiro, José Mauro da Cunha e Castro, Maura Da Cunha, Vanildo Silveira, Antônia Elenir Amâncio Oliveira, Kátia Valevski Sales Fernandes

**Affiliations:** ^1^ Laboratório De Química e Função De Proteínas e Peptídeos Universidade Estadual do Norte Fluminense Darcy Ribeiro Rio de Janeiro Brazil; ^2^ Laboratório De Biotecnologia Universidade Estadual do Norte Fluminense Darcy Ribeiro Rio de Janeiro Brazil; ^3^ Laboratório De Biologia Celular e Tecidual Universidade Estadual do Norte Fluminense Darcy Ribeiro Rio de Janeiro Brazil; ^4^ Empresa Brasileira de Pesquisa Agropecuária Semiárido Petrolina Pernambuco Brazil; ^5^ Empresa Brasileira de Pesquisa Agropecuária Soja Londrina Paraná Brazil

**Keywords:** crop resistance, guava (*Psidium guajava*), immune mechanisms, plant‐parasitic nematodes, root anatomy

## Abstract

Guava (*Psidium guajava*), referred to as the “tropical apple,” is esteemed for its sweet flavor, nutritional density, and medicinal attributes, being rich in ascorbic acid, phenolics, carotenoids, fibers, and minerals. Despite its agricultural significance, guava cultivation faces considerable challenges from plant‐parasitic nematodes, particularly root‐knot nematodes from the *Meloidogyne* spp. In South America, *Meloidogyne enterolobii* causes severe root damage and economic losses to this crop. Plants fight nematodes through complex immune mechanisms involving pattern recognition receptors and signaling pathways, such as pattern‐triggered immunity. The present research employed comparative shotgun proteomic analysis complemented by microscopic imaging and histochemical assays of roots from susceptible *P. guajava* and resistant *P. guineense*, inoculated or not with *M. enterolobii. Psidium‐M. enterolobii* interactions revealed intricate plant cellular responses such as giant cells formation, hypersensitivity reactions, and biochemical pathway adjustments in sucrose transport and antioxidant enzyme activities. Synthesis and accumulation of secondary metabolites like terpenes, alkaloids, and phenolics in inoculated and resistant plants were positively correlated to plant resilience. Heat shock proteins and protein disulfide isomerases also emerged as pivotal in plant response, being upregulated during nematode infection.

**Summary:**

- The work addresses and unravels some of the puzzle pieces in the net of processes triggered in a plant prey (*Psidium* spp.), of either susceptible (*P. guajava*) or resistant (*P. guineense*) phenotypes, when confronted by its nematode predator (*Meloidogyne enterolobii*).
- The main alterations detected in the roots of these plants ranged from giant cells formation, hypersensitivity reactions, biochemical adjustments in sucrose transport pathways and in antioxidant enzyme activities, to increases in secondary metabolites (terpenes, alkaloids, and phenolics) and in heat shock proteins and protein disulfide isomerases.
- All these defensive mechanisms were triggered by the nematode attack on both species and were more prominent in *P. guineense*, which positively correlates them to the plant resistance against *M. enterolobii*.

## 1 Introduction

The guava (*Psidium guajava)*, known as the “tropical apple,” is a fruit belonging to the Myrtaceae family cultivated in various regions, including India, Pakistan, and South America. In 2024, global production reached approximately 63.1 million tons [1] and guava market size is estimated to reach US$1250 million by 2027 [2]. A sweet, tasty fruit, it also bears nutritive and therapeutic properties. Various parts of the guava tree, such as roots, leaves, bark, stem, and fruits, are utilized in treatments of respiratory and gastrointestinal disorders [3]. Contemporary research has identified phenolic compounds and terpenes as bioactive constituents of guava leaf extract, abundant in antioxidants, and employed as a food preservative and medication [4].

However, there is a problematic challenge in guava cultivation which is the frequent infestation of its roots by nematodes. It is estimated that plant‐parasitic nematodes (PPNs) cause over 100 billion dollars in crop losses annually, with approximately US$500 million spent on PPNs management each year. Root‐knot nematodes (RKNs), such as those from the *Meloidogyne* genus, are sedentary endoparasites and represent one of the economically important PPNs. The females deposit up to 1000 eggs near the roots, forming a gelatinous mass. Second‐stage juveniles invade the root tip, migrating through the vascular system. RKNs become sedentary at feeding sites, inducing the formation of giant cells for their development [5]. *M. enterolobii*, known as the guava RKN, poses a global threat to this fruit culture and is highly virulent even to plants resistant to other nematode species [6]. Inflicting significant damage to guava trees in South America, it can lead to losses exceeding 65%, often detected only post‐harvest when a high number of galls is observed in the roots [6, 7].

Plants exhibit complex immune systems against nematodes, producing anti‐nematode molecules either constitutively or post‐infection. Plant resistance encompasses the ability to restrict the growth and reproduction of pests or pathogens and can be qualitative, preventing reproduction entirely, or quantitative, reducing the severity of the disease [8]. Over the course of evolution, plants have evolved defense mechanisms to counteract the invasion of gall‐forming nematodes [9]. The signaling process begins with the nematode chemotaxis toward the root, attracted by exudates. Signaling takes place through nematode‐associated molecular patterns (NAMPs) recognized by plant pattern‐triggered immunity (PTI) [10]. PTI results in gene expression, production of reactive oxygen species, and physical responses. However, pathogens can overcome this defense through effectors, suppressing PTI by inhibiting proteins or altering their active state [9].

Progress in genomics, proteomics, natural product chemistry and biotechnology represents a potential avenue for enhancing our comprehension of root‐nematode interactions and translating this information into environmentally sound management strategies. The current study aimed to conduct a comparative shotgun proteomic analysis of the roots from *P. guajava* (susceptible to *M. enterolobii*) and *P. guineense* (resistant to this nematode), both inoculated and non‐inoculated by this pathogen. To observe modifications in root anatomy resulting from infection, microscopic images were recorded, and histochemical assays were conducted to evaluate secondary metabolites, such as phenolic compounds, terpenes, and alkaloids.

## 2 Materials and Methods

### 2.1 Biological Material

*P. guajava* and *P. guineense* were germinated from seeds in a greenhouse at Embrapa Semiárido, in Petrolina, PE (Latitude: 9°09'S, longitude: 40°22'W, altitude: 365.5 m), using appropriate cultural practices. Thirty days after planting, seedlings were inoculated with a suspension containing 10,000 eggs and second‐stage juvenile nematodes. Inoculation was performed by applying the suspension into two holes, each 2 cm deep in the soil, at 2 cm from the seedling stem. After inoculation, the holes were filled with soil, and a light irrigation was carried out.

Root samples were collected 20 days after inoculation (20 DAI), for microscopy analysis. At this time, the oviposition activity of the nematodes was at its peak under our environmental conditions. The samples were washed in running water to remove the substrate. Samples without inoculation were collected as a control for the study (20 days non‐inoculated [DNI]).

For proteomic analyses, 30 roots were collected from each test condition, at 5 and 20 DAI as well as other 30 without inoculation (20 DNI). All samples were washed in running water and lyophilized, and three groups of 10 roots for each species were prepared for each test condition. The material was crushed, pulverized in liquid nitrogen using a mortar and pestle, and stored at—4°C in Falcon tubes.

### 2.2 Light, Transmission Electron, and Scanning Electron Microscopy

The root samples were placed at room temperature in 2.5% glutaraldehyde, 4.0% formaldehyde, and 0.05 M sodium cacodylate buffer with pH 7.2 for 2 h, and then washed three times for 30 min each in the same buffer, before post‐fixed for 1 h in a solution of 1% osmium tetroxide and 1 M sodium cacodylate buffer with pH 7.2. After three 30 min washes in the same buffer, the samples were subjected to a series of acetone dehydration steps: 30%, 50%, 70%, 90%, 100%, and two times 100% super dry. After the fixation and dehydration steps, epoxy resin (Epon 812) was gradually infiltrated to replace acetone. The resin‐impregnated samples were placed in molds and polymerized in an oven at 60°C for 48 h. Semi‐thin sections between 0.60 and 0.70 µm were obtained using an ultramicrotome (Reichert Ultracuts) and a diamond knife.

#### 2.2.1 Light Microscopy (LM)

The 0.60–0.70 µm sections were mounted on slides and stained with 1% toluidine blue for 1 min [11]. After sealing the slides with Entellan, the samples were analyzed using a bright‐field light microscope Axioplan (ZEISS, USA) coupled with a Canon PowerShot A640 camera. The images were used for qualitative description.

#### 2.2.2 Transmission Electron Microscopy (TEM)

After infiltration and embedding steps, the 0.60–0.70 µm sections were collected on 300 mesh copper grids and contrasted with 5% uranyl acetate for 40 min and 1% lead citrate for 5 min, at room temperature [11]. The sections were then observed using a JEOL TEM 1400 PLUS microscope.

#### 2.2.3 Scanning Electron Microscopy (SEM)

After the acetone dehydration steps, the root samples underwent critical point drying, using the Bal‐tec Critical Point Dryer CPD 030 equipment. The fragments were mounted on appropriate supports using carbon adhesive tape and coated with a thin layer of 20 nm gold using the Bal‐tec Sputter Coater SCD 050. The samples were observed under a EVO40‐ZEISS microscope, at an acceleration of 25 kV.

### 2.3 Histochemical Tests: Phenolic Compounds, Terpenes, and Alkaloids

The fixed and washed root samples were dehydrated in an alcohol series: 50%, 70%, 90%, and 100% and infiltrated and embedded in Historesin (Leica Instruments, Wetzlar, Germany). Using a disposable blade, thin sections of 4 µm thickness were obtained at a rotary microtome (Cut 4050 Slee Mainz).

The sections were exposed to specific reagents and mounted between a slide and coverslip with 50% glycerin. For phenolic compounds detection, sections were treated with a 10% ferric chloride solution in water for 30 min, and quickly rinsed with water. Total phenolic compounds stain from brown to black [12]. For terpenes, sections were incubated for 1 h in the dark in NADI solution (0.5 mL of 0.1% alpha‐naphthol in 40% ethanol; 0.5 mL of 1% dimethyl‐p‐phenylenediamine hydrochloride in water; and 49 mL of 0.05 M sodium phosphate buffer pH 7.2). Then, they were washed for 2 min with 0.1 M sodium phosphate buffer pH 7.2. Terpenes stain from blue (essential oils) to red (acidic resins) and acquire a violet to purple coloration when there is a mixture of these substances [13]. For alkaloids, sections were incubated for 10 min in Wagner's reagent (0.01 M potassium iodide and 0.01 M iodine in 100 mL of water) and quickly rinsed with water. Alkaloids stain reddish‐brown [14].

All microscopic preparations were examined and captured under a bright‐field light microscope Axioplan (ZEISS, USA) coupled with a Canon PowerShot A640 camera. The images were used for qualitative description.

### 2.4 Comparative Shotgun Proteomic Analysis

#### 2.4.1 Total Protein Extraction and Quantification

Pulverized roots (500 mg) were divided into two 2 mL microtubes. To each microtube, 1.5 mL of urea/thiourea extraction buffer (7 M urea, 2 M thiourea, 1 mM PMSF in ethanol, 65 mM DTT, and 2% Triton X‐100) was added. The tubes were constantly shaken at 4°C for 30 min and incubated at –20°C for 30 min before centrifuged at 16,000 *g* at 4°C for 20 min. Protein concentration of the resulting supernatants was estimated using the 2‐D Quant Kit (Cytiva, Marlborough, MA, USA).

#### 2.4.2 Precipitation and Protein Digestion

Before the trypsin digestion step, aliquots of 100 µg of proteins were precipitated using the methanol/chloroform methodology to remove any contaminants from the samples [15]. Each biological replicate was then digested using a Microcon‐30 kDa filter (Millipore) with the Filter‐Aided Sample Preparation (FASP) methodology [16] with modifications [17], at a trypsin‐to‐protein ratio of 1:100 (V5111, Promega).

Obtained peptides were vacuum‐dried, solubilized in 0.7 µg/µL in 95% water and 5% acetonitrile plus 0.1% formic acid and quantified at a NanoDrop 2000c spectrophotometer (Thermo Fisher Scientific, Waltham, USA) for mass spectrometry (MS).

#### 2.4.3 Mass Spectrometry Analysis

Mass spectrometry was performed using a nanoACQUITY Ultra Performance Liquid Chromatography (UPLC) system coupled to a Q‐TOF SYNAPT G2‐Si instrument (Waters, Manchester, United Kingdom) [18] and details of procedures and conditions are compiled in Supporting Information S1.

#### 2.4.4 Proteomic Data Analysis

The spectral processing and database search conditions were performed using ProteinLynx Global SERVER (PLGS) software (version 3.02, Waters). The parameters for HDMSE analysis are in Supporting Information S1. The proteome of the species *Eucalyptus grandis* (ID: UP000030711), deposited in UniProtKB (https://www.uniprot.org/), was used as the reference proteome, because it belongs to the same botanical family of Myrtaceae and is phylogenetically the closest relative to *Psidium* spp. with proteomic data available.

The label‐free quantification analysis was performed using ISOQuant software v.1.8 [19, 20], with parameters described in Supporting Information S1. For comparative analysis, only proteins present or absent (for unique proteins) in the three biological replicates were accepted for differential abundance analysis. The following comparisons were performed: GUA_20DAI/GUA_20DNI; GUA_20DAI/GUA_5DAI; GUI_20DAI/GUI_20DNI; GUI_20DAI/GUI_5DAI (GUA stands for *P. guajava* and GUI stands for *P. guineense*). The data were analyzed by the two‐tailed Student's *t*‐test. Proteins with a *p* value < 0.05 were considered increased if the Log2 fold change (FC) was >0.6 and decreased if the Log2 FC was <–0.6.

From the list of proteins between the six conditions tested, a principal component analysis (PCA) was performed using the MetaboAnalyst 6.0 program (https://www.metaboanalyst.ca/). Volcano plots were generated with GraphPad Prism 6 for Windows program, GraphPad Software, Boston, Massachusetts, USA (www.graphpad.com) to identify proteins with increased and decreased accumulation over 20 days, with and without inoculation of *M. enterolobii*, for the two species studied.

Proteins identified as upregulated or downregulated over 20 days were grouped into sets using a Venn Diagram (https://bioinformatics.psb.ugent.be/webtools/Venn/). Interaction networks of the differentially abundant proteins (DAPs) were constructed considering the first interaction level retrieved from STRING version 12.0 (https://string‐db.org). To generate a protein‐protein interaction (PPI) network, *E. grandis* was the reference plant species, using a minimum interaction score of 0.7 through Cytoscape, version 3.10.1 (https://cytoscape.org). After identifying the upregulated and downregulated groups for each species, heatmaps were generated for each cluster using Heatmapper (http://www.heatmapper.ca/).

## 3 Results and Discussion

### 3.1 Microscopy Images and Qualitative Analysis of Secondary Metabolites From the Roots of *Psidium* spp. Inoculated and Not Inoculated by *M. enterolobii*

To initially confirm the identification of the nematode structure present in the plant vascular cylinder, transmission electron microscopy images of the pathogen extracted from the inoculum used in this study were acquired (Figure S1).

**FIGURE 1:**
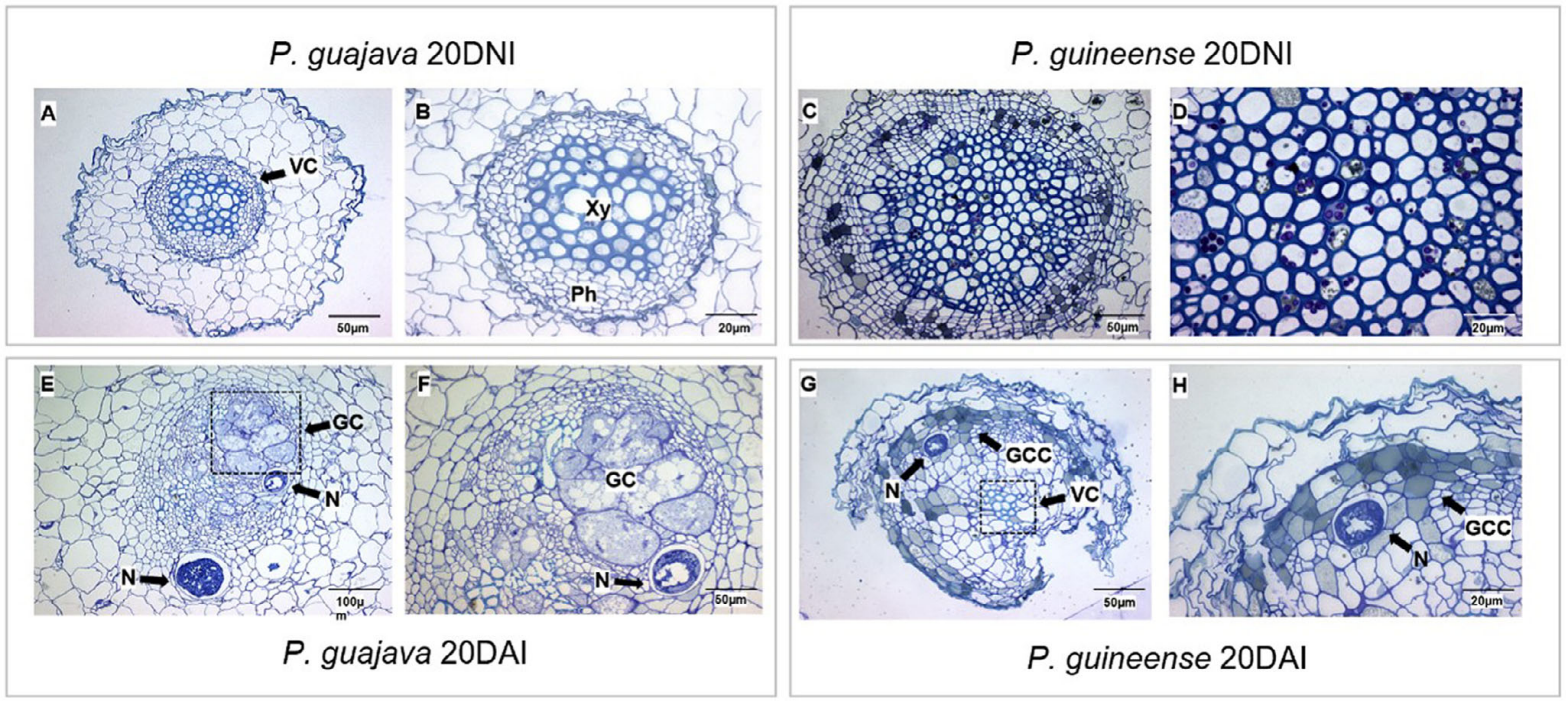
Images of the roots obtained by bright field light microscopy: GUA_20DNI (A and B); GUI_20DNI (C and D); GUA_20DAI (E and F) e; GUI_20DAI (G and H). *P. guajava*—GUA; *P. guineense*—GUI; 20 days non‐inoculated—20DNI; 20 days after inoculation—20DAI; Vascular cylinder—VC; Xylem–Xy; Phloem—Ph; Giant cells—GC; Nematode—N; Grayish‐colored cells—GCC.

Figure 1 presents images of transverse root sections acquired through bright‐field light microscopy. In 20 days, samples of both *P. guajava* and *P. guineense* non‐inoculated with *M. enterolobii* (GUA and GUI_20DNI, respectively), the structure of the root vascular cylinder exhibits general organization without alteration (Figure 1A–D). In inoculated *P. guajava* (GUA_20DAI) (Figure 1E,F), the presence of the nematode along with feeding sites containing multinucleated giant cells (GC) indicates successful infection in the host plant. In the case of *P. guineense* after 20 days of inoculation (GUI_20DAI) (Figure 1G,H), this organization of vascular tissues is no longer evident, and a reduction of the vascular cylinder is mainly noted. The presence of the nematode in the peripheral region of the cylinder is observed, accompanied by the appearance of grayish‐colored cells, suggesting the formation of compounds associated with the presence of the phytopathogen. The tissue disorganization and a reduction in the vascular cylinder of the resistant phenotype, where no GC were seen, suggests that these structures are essential for infection and the formation of the pathosystem.

The formation of giant cells (GC) and the hypersensitivity response (HR) are plant defense mechanisms during pathogen invasion [21]. A histopathological study of okra (*Abelmoschus esculentus*) roots revealed that second‐stage juveniles of *M. incognita* were able to feed and reproduce due to the formation of GC [22]. The accelerated metabolic activity at these feeding sites causes nutrient deficiency in plant tissues, leading to systemic responses throughout the organism [23]. GCs are crucial for the establishment of the nematode in the vascular cylinder, and such location is strategic. According to Xu et al. [24], sucrose transport through plasmodesmata leads to an efficient nutrient distribution through the plant vascular system, supplying energy to regulate plant metabolism. In this context, pectins are essential in the cell wall, promoting adhesion and porosity, with emphasis on homogalacturonans, whose methylesterification is associated with nematode‐induced lesions and gall formation, disrupting nutrient transport and altering cell structure and function [25]. Infection can modify pectic polysaccharides, affecting cell expansion and nutrient absorption [26]. The endodermis, with walls reinforced by suberin and lignified Casparian strips (CS), protects the vascular cylinder against biotic and abiotic threats. Studies in *Arabidopsis* with defects in CS have shown an impact on the infectivity of *M. incognita* and on the size of the feeding site [27]. After infection by *Meloidogyne* spp., the expression of several cell wall‐modifying enzymes, such as expansins, endoglucanases, extensins, hydrolases, and structural proteins, was altered [28].

**FIGURE 2:**
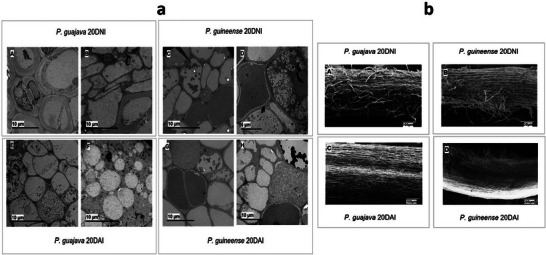
Images of roots obtained by transmission electron microscopy (a)—GUA_20DNI (A and B); GUI_20DNI (C and D); GUA_20DAI (E and F); and GUI_20DAI (G and H) and scanning electron microscopy (b)—GUA_20DNI (A); GUI_20DNI (B); GUA_20DAI (C); and GUI_20DAI (D). *P. guajava*—GUA; *P. guineense—*GUI; 20 days non‐inoculated—20DNI; 20 days after inoculation—20DAI.

To investigate cell morphology, images of root sections were examined by TEM (Figure 2a). For *P. guajava*, at the GUA_20DNI condition, Figure 2aA displays the xylem, and Figure 2aB, the organized xylem and pericycle. At the GUA_20DAI condition, Figure 2aE reveals the central cylinder with substance accumulation, and Figure 2aF shows multinucleated cells indicating the development of feeding sites of *M. enterolobii*. In contrast, in *P. guineense*, there is no change in cell wall thickness when comparing the conditions with and without nematode inoculation. It is observed that, in *P. guineense*, high concentrations of substances are present in both conditions (Figure 2aC–aH), but the density of these substances increases in response to the pathogen (Figures 2aG,aH).

From the SEM images (Figure 2b), changes in the surface layer of the roots were observed after inoculation with *M. enterolobii* in both species. In *P. guajava*, at the non‐inoculated condition (GUA_20DNI) (Figure 2bA), root hairs, which play a crucial role in the absorption of water and nutrients by the plant, can be seen. After inoculation (GUA_20DAI) (Figure 2bC), reduction and morphological alterations of root hairs were seen. In *P. guineense* (GUI_20DAI) (Figure 2bD), there is also a reduction in root hairs, although they are still present, with slight peeling of the outermost layer of the root compared to the control (GUI_20DNI) (Figure 2bB).

Root‐knot nematodes inhibit plant growth by reducing the root system and decreasing water and nutrient absorption, resulting in water stress and nutritional deficiency [29]. Phytohormones are crucial as signaling molecules in root hair growth and development. Auxin and ethylene, in particular, play vital roles in this regulation, interacting with jasmonic acid, abscisic acid, strigolactones, and brassinosteroids [30]. Our following data on changes in secondary metabolites also suggest that these may affect the production of phytohormones essential for maintaining root hairs.

**FIGURE 3:**
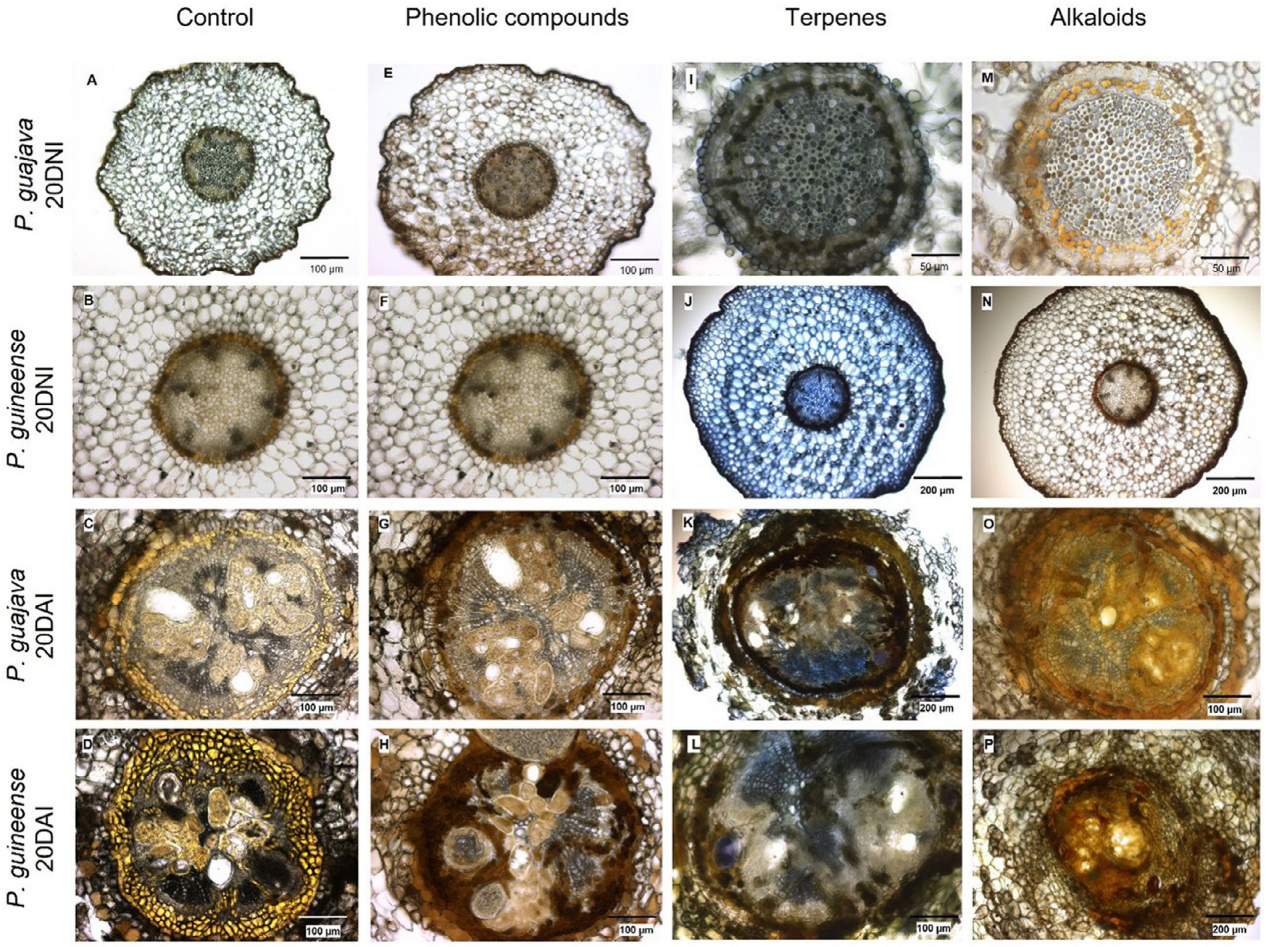
Images obtained by light field optical microscopy after staining for histochemical testing of *Psidium* roots: control roots of *P. guajava* (non‐inoculated with *M. enterolobii*)—GUA_20DNI (row 1), control roots of *P. guineense* (non‐inoculated)—GUI_20DNI (row 2), *P. guajava* roots 20 days after inoculation—GUA_20DAI (row 3) and *P. guineense* roots 20 days after inoculation—GUI_20DAI (row 4): control (column 1); phenolic compounds stained with ferric chloride (column 2); terpenes stained with NADI solution (column 3); and alkaloids stained with Wagner's Reagent (column 4).

A homogeneous distribution of phenolic compounds from the epidermis to the endodermis in both species (GUA and GUI_20DNI) was observed under non‐inoculated conditions (Figure 3E,F). However, after inoculation (GUA and GUI_20DAI), besides an increased diameter of the root intersection, there was an increase in the production of these compounds (Figure 3G,H). In *P. guajava*, this increase is more notable in the endodermis, while in *P. guineense*, this trend extends to the central areas of the cylinder. A significant presence of terpenes was observed throughout the structure of *P. guineense* inoculated roots compared to the control condition of both species (GUA and GUI_20DNI) (Figure 3I,J). After inoculation (GUA and GUI_20DAI), there was an amplification of terpenes staining (Figure 3K,L). Alkaloids were seen throughout the structure of control roots of *P. guajava* (Figure 3M) compared to the control roots of *P. guineense* (Figure 3M,N, respectively). After inoculation (GUA and GUI_20DAI), a pronounced increase is observed in both species (Figure 3O,P).

Our histochemical analysis, in summary, showed increases in root diameter and in the production of phenolic compounds, terpenes, and alkaloids in both *P. guajava* and *P. guineense* after inoculation with *M. enterolobii*. In the roots of *P. guineense*, there was a greater accumulation of these compounds, suggesting an efficient activation of defense responses by the species. A hypersensitive reaction in the root cortex of *Oryza glumaepatula* was similarly recorded two days after inoculation with *M. graminicola*, when few nematode juveniles were established in the central cylinder, with rare collapsed giant cells surrounded by degenerated female bodies displaying accumulation of phenolic compounds [31].

Secondary metabolites, such as phenolic compounds, terpenes, and alkaloids, possess various properties, including antifungal, antibacterial, antioxidant, and insecticidal activities [32]. Phenolic compounds, synthesized through shikimic acid and phenypropanoid pathways, are essential for various plant functions, including cell wall thickening, hormonal synthesis, pigmentation, and defense against pathogens [33, 34]. The role of the shikimic acid pathway in nematode infection, as observed in *P. guineense* and *P. cattleianum* infected by *M. enterolobii*, highlights the importance of tannins and hydrolysable lignans in the protection of infected tissues [35]. Increases in hydrogen peroxide, superoxide dismutase activity, and catalase in pumpkins [36] and the stimulation of phenylpropanoid biosynthesis in watermelons [37], both infected by *M. incognita*, were reported. In guava, after infection by *M. enterolobii*, increases in phenols, proline, peroxidase, and polyphenol oxidase were observed [38]. These findings highlight the dynamic interaction between plants and nematodes, mobilizing antioxidant defenses against infectious stress. Terpenes are essential in plant interactions with soil organisms [39], contributing to the synthesis of hormone‐induced defense molecules such as salicylic acid, jasmonic acid, and ethylene [40]. Non‐host Asteraceae plants show potential as nematode repellents through compounds like (E)‐β‐farnesene and 1,8‐cineole, highlighting the role of terpenoids in nematode control [41]. Pyrrolizidine alkaloids (PAs) from *Crotalaria* spp. exhibited nematotoxicity, with field tests showing significant reductions in nematode populations [42]. Tryptophan acts as a precursor for indole alkaloids, phytoalexins, and glucosinolates, which are essential in plant defense against microbial infections and herbivore attacks, including nematicidal activity [43, 44].

### 3.2 Comparative Proteomic Analysis of the Roots of *Psidium* spp. Inoculated and Non‐Inoculated

**FIGURE 4:**
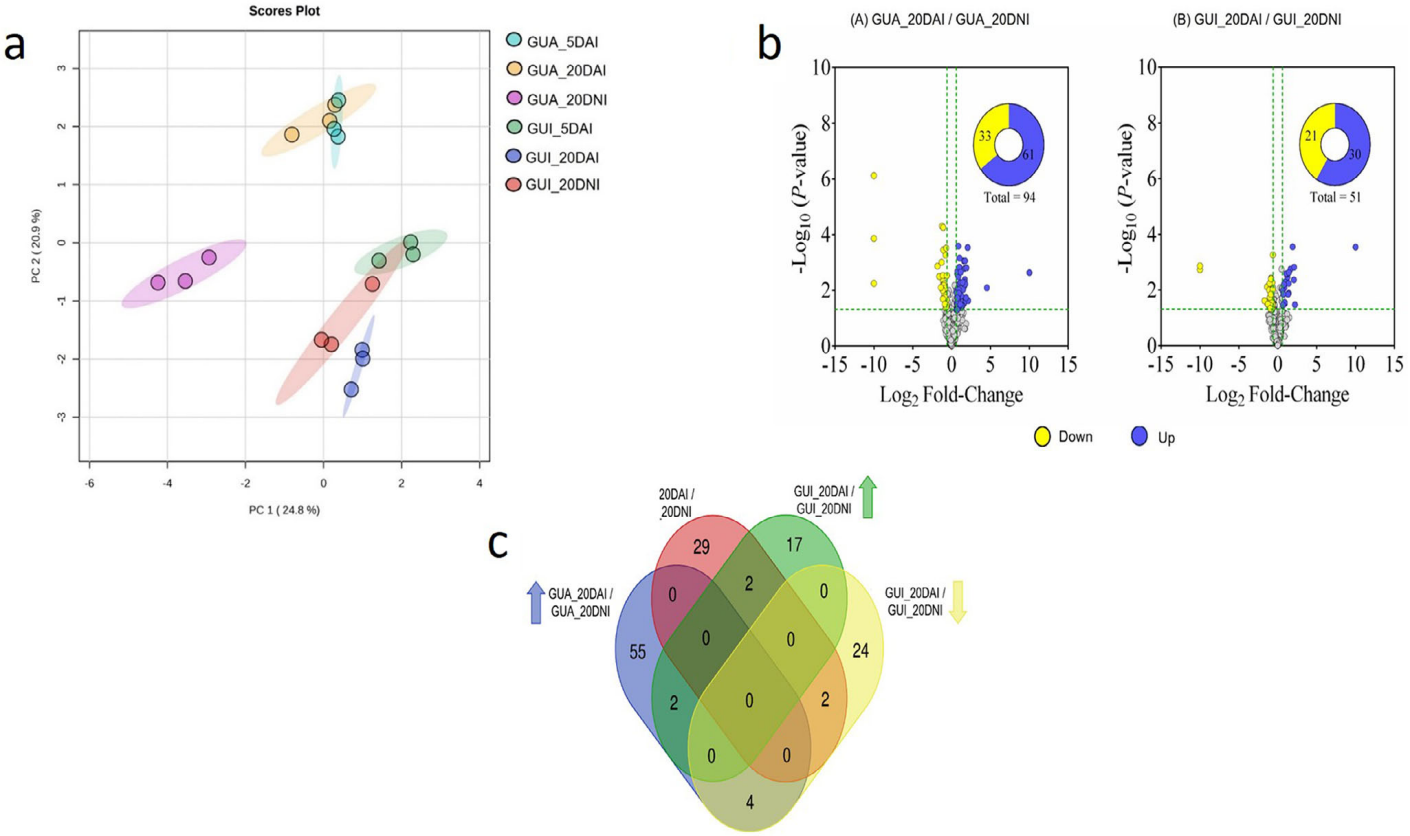
Multivariate analysis of principal components for the set of 416 selected proteins from roots of *Psidium*. (a) [*P. guajava* 5 days after inoculation‐ GUA_5DAI; *P. guajava* 20 days after inoculation—GUA_20DAI; *P. guajava* after 20 days non‐inoculation of *M. enterolobii*—GUA_20DNI; *P. guineense* 5 days after inoculation—GUI_5DAI; *P. guineense* 20 days after inoculation—GUI_20DAI; *P. guineense* after 20 days non‐inoculation—GUI_20DNI], Volcano distribution plot of the up‐ and down‐regulated proteins among the whole 416 protein set from *P. guajava* and *P. guineense* samples (b) [(A) GUA_20DAI / GUA_20DNI—comparison between *P. guajava* 20 days after inoculation and *P. guajava* 20 days after non‐inoculation with *M. enterolobii*; (B) GUI_20DAI/GUI_20DNI—comparison between *P. guineense* 20 days after inoculation and *P. guineense* after 20 days of non‐inoculation with the nematode; gray circles—unchanged], and a Venn diagram of 135 differentially accumulated proteins (DAPs) after 20 days. (c) [GUA_20DAI/GUA_20DNI—comparison between *P. guajava* 20 days after inoculation and *P. guajava* 20 days after non‐inoculation with *M. enterolobii*, up (blue) and down‐regulated (pink); GUI_20DAI/GUI_20DNI—comparison between *P. guineense* 20 days after inoculation and *P. guineense* after 20 days after non‐inoculation with *M. enterolobii*, up (green) and down‐regulated (yellow).

Based on statistical criteria, 416 proteins were identified and selected between the treatments. Figure 4a shows a multivariate principal component analysis (PCA), allowing observation of the behavior of the groups of individuals. According to the PCA for *P. guajava*, the GUA_20DNI set forms a distinct space, without overlapping with the samples from the condition after 5 and 20 days of inoculation. Notably, there is overlap between part of the GUA_20DAI set and the universe of GUA_5DAI. In the case of *P. guineense*, it stands out for the proximity of control individuals GUI_20DNI with those inoculated after 5 days and after 20 days. Additionally, it is relevant to observe that individuals GUA_20DAI occupy an opposite position in relation to individuals GUI_20DAI.

The Volcano plot (Figure 4b) represents the changes at the levels of the 416 proteins from *P. guajava* and *P. guineense* samples, classified as up‐ and down‐regulated at the 20‐day time point, in presence or absence of nematode. In *P. guajava*, inoculation led to an increase of 61 proteins (14.7%) and a decrease of 33 proteins (7.9%) in protein expression, with 69.4% of the proteins showing no changes in their accumulation (Figure 4bA). In *P. guineense*, values were 7.2% (30 proteins) and 5% (21 proteins) for up‐ and down‐regulated proteins, respectively, and 82% of unaltered proteins (Figure 4bB).

From the 145 proteins with altered expression in both species, 135 proteins were increased or decreased exclusively in one or other species and condition, with the remaining 10 proteins being shared among them. This can be observed by the Venn Diagram (Figure 4c), where clusters generated by the proteins are discriminated.

Among the analyzed proteins, the following were up‐regulated in both species: A0A059DIT8 (Ribosomal protein S7 domain‐containing protein) and A0A058ZUU6 (5‐methyltetrahydropteroyltriglutamate–homocysteine S‐methyltransferase). On the other hand, the proteins A0A059AZ85 (isopentenyl‐diphosphate delta‐isomerase) and A0A059D4J5 (L‐ascorbate peroxidase), related to the biosynthetic process of dimethylallyl diphosphate, an intermediate metabolite of the mevalonate pathway, were down‐regulated in both species. Some proteins showed different patterns between the two species. For example, A0A059AGF2 (related to NADP+‐dependent isocitrate dehydrogenase (IDH) activity), A0A058ZSX3 (Bet v I/Major latex protein domain‐containing protein), A0A059B9 × 7 (Coatomer subunit gamma), and A0A059A4C7 (UDP‐arabinopyranose mutase) were up‐regulated in *P. guajava* but down‐regulated in *P. guineense*. Meanwhile, A0A059DGU6 (14‐3‐3 domain‐containing protein) and A0A059CN57 (NAD_binding_2 domain‐containing protein) showed the opposite pattern, being down‐regulated in *P. guajava* and up‐regulated in *P. guineense*. In *P. guajava*, it was observed that 55 DAPs increased, while 29 DAPs decreased when comparing the conditions of 20 days post‐non‐inoculation and inoculation (Figure 4c). On the other hand, in *P. guineense*, 17 DAPs were up‐regulated and 24 DAPs down‐regulated when comparing the same conditions (Figure 4c). The enrichment of the groups of up‐ and down‐regulated proteins in each species was analyzed.

**FIGURE 5:**
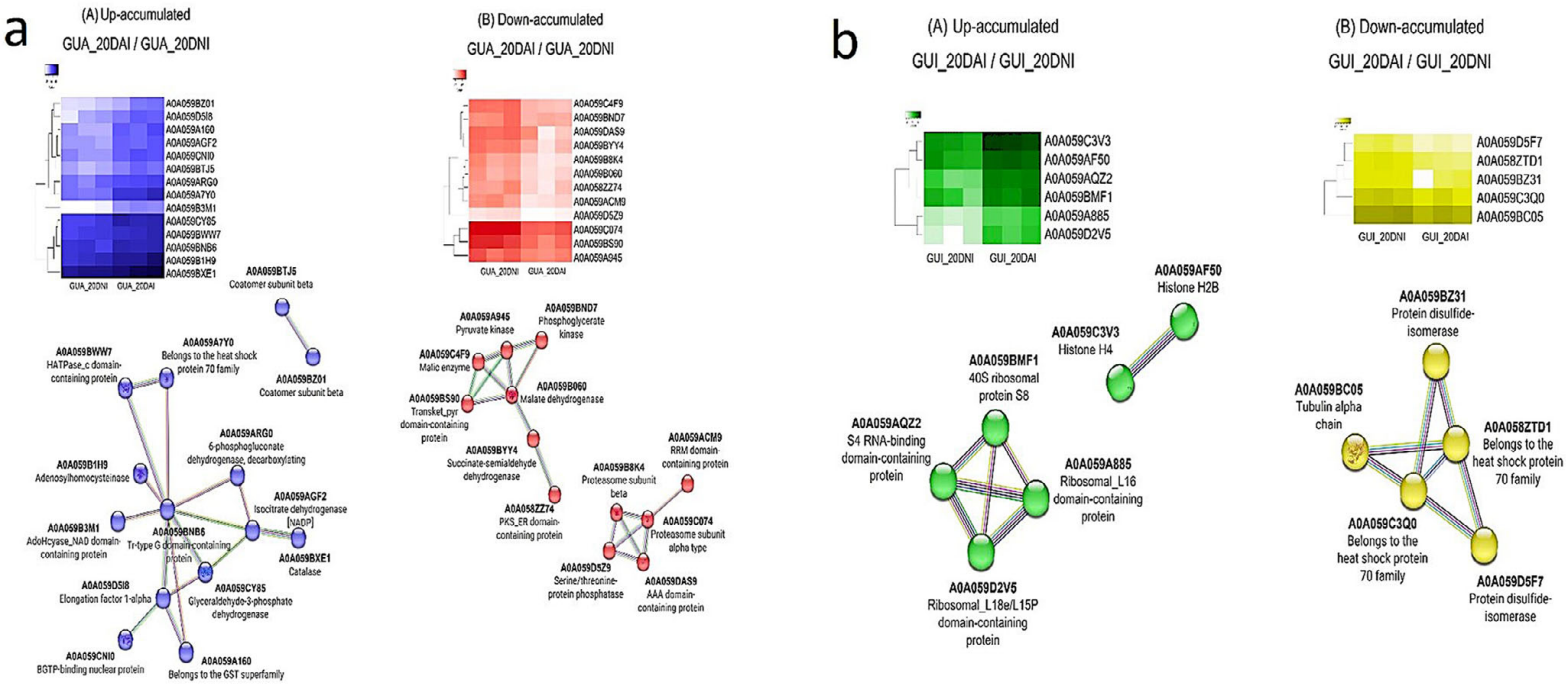
Heatmaps together with interaction networks showing the up (A) and down (B) regulated proteins, comparing *P. guajava* 20 DAI and non‐inoculated with *M. enterolobii* (GUA_20DAI/GUA_20DNI) (a) and *P. guineense* 20 DAI and non‐inoculation with *M. enterolobii* (GUI_20DAI/GUI_20DNI) (b).

In Figure 5a, based on minimum required interaction score criteria, interaction networks among the identified proteins were established for *P. guajava* (GUA_20DAI/GUA_20DNI, 14 up‐regulated proteins, 12 down‐regulated proteins—total of 26 proteins). In Figure 5b, the representation for the networks of *P. guineense* is shown (GUI_20DAI/GUI_20DNI, six up‐regulated proteins and five down‐regulated proteins—total of 11).

When investigating the behavior of proteins after 5 days of nematode inoculation and comparing with the data after 20 DAI, volcano plots were generated for each species in the study (Figure S2). In *P. guajava*, out of the 26 analyzed proteins, only one (A0A059AGF2, isocitrate dehydrogenase [NADP]) was accumulated from 5 to 20 DAI, while the others did not show significant changes according to statistical analysis (Figure S2A). On the other hand, in *P. guineense*, all 11 proteins remained unchanged, suggesting that changes in plant metabolism mainly occur within the first 5 DAI and persist in the subsequent days (Figure S2B).

Based on the protein networks, it was observed that *P. guajava* exhibits an increased accumulation of proteins associated with the activities of catalase (CAT), adenosylhomocysteinase, and hydrolase 20 DAI of *M. enterolobii*. This suggests that these pathways may be related to the infection process caused by the nematode. When stressed, plants activate signaling molecules, such as ROS. The invasion of pathogens disrupts this production, causing oxidative damage to lipids, proteins, and nucleic acids [45]. Catalase and ascorbate oxidase are essential in antioxidant defense, neutralizing ROS before they cause harm. During nematode infections, the activity of hydrogen peroxide and catalase increases rapidly [46]. In a study with *M. incognita*, the enzymes CAT, superoxide dismutase (SOD), guaiacol peroxidase (GuPOX), ascorbate peroxidase (APOX), polyphenol oxidase (PPO), glutathione S‐transferase (GST), and non‐enzymatic antioxidants increased in tomatoes under nematode stress [47]. CAT is inhibited in resistance responses but increases in successful infections [48].

In the last decade, studies on epigenetic regulators revealed how these mechanisms influence plant‐nematode interactions. While plants respond rapidly to infection, nematodes can manipulate epigenetic modifications to suppress plant defenses [49]. The balance between histone acetyltransferases and histone deacetylases controls histone acetylation, influencing gene expression and biological processes [50, 51]. On the other hand, histone hypoacetylation promotes gene silencing [52, 53]. In *P. guineense*, a significant increase in histone proteins H4 and H2B is observed 20 DAI with *M. enterolobii*, suggesting they are related to resistance against nematode infection. Studies indicate that histone‐modifying enzymes are deregulated in nematode‐induced galls in rice, suggesting a dynamic response of histone modifications during RKN infection [50]. The interaction between rice and *M. graminicola* revealed different expression profiles of components of the epigenetic machinery, reinforcing that DNA hypomethylation and changes in histone acetylation and methylation are part of the immune response against RKN [54].

Disulfide isomerase (PDI) was negatively regulated in *P. guineense* 20 DAI with *M. enterolobii*. Protein disulfide isomerases (PDI), found in the endoplasmic reticulum (ER), catalyze the formation, breaking, and rearrangement of disulfide bonds between cysteine residues in proteins [55]. In pathogens, they play essential roles in protecting them against ROS released by hosts during infection [56]. Recent studies have characterized nematode effectors, such as PDIs, that induce cell death in plants and contribute to parasitism. For example, MiPDI1 from *M. incognita* increases *Arabidopsis* susceptibility to the nematode [57].

Under inoculation with *M. enterolobii*, *P. guajava* showed an increase in the accumulation of a 70 kDa heat shock protein, while *P. guineense* experienced a decrease. The HSP70s regulate stress responses and activate the plant immune system against infections. Deficiency in HSP70s, compared to other HSPs, results in plant growth retardation, especially under stress conditions [58]. After stress relief, HSPs return to nearly normal levels through mechanisms that are not yet fully understood, involving a transient reprogramming of cellular activities and specific induction of molecular chaperones [59]. The GmHsp22.4 gene, strongly induced in soybean lines resistant to *M. javanica* but repressed in susceptible ones, reduced nematode reproduction by up to 82% in transgenic *Arabidopsis* [60]. The heat shock proteins HSP‐4, HSP90 and HSP20 were also seen to be accumulated in *Vigna unguiculata* infected by *M. incognita* [61].

Proteomic and transcriptomic analyses have shown that plants respond to nematode stress by accumulating various proteins, such as HSPs and PDIs, as well as other proteins related to defense and detoxification. These studies are essential for developing integrated management strategies and genetically improving plant resistance to these pathogens [62, 63].

## 4 Associated Data

The datasets generated during and/or analyzed during the current study were deposited in the ProteomeXchange Consortium via the PRIDE partner repository [64] with the dataset identifier PXD058189.

## 5 Conclusions

*Psidium* responses to *M. enterolobii* involve a complex interplay of cellular processes, including giant cell formation, hypersensitivity responses, and alterations in biochemical pathways such as sucrose transport, pectin modification, and antioxidant enzyme activity. These responses are reinforced by the synthesis and accumulation of secondary metabolites like terpenes, alkaloids, and phenolic compounds, contributing to plant resilience. Moreover, epigenetic regulation through histone modifications and the role of molecular chaperones such as heat shock proteins and protein disulfide isomerases are also undertaken as adaptive strategies that inoculated plants employ to combat nematode infections.

## Conflicts of Interest

The authors have no relevant financial or non‐financial interests to disclose.

## Supporting information




**Supporting Information file 1**: pmic70015‐sup‐0001‐SuppMat.docx

## Data Availability

The data that support the findings of this study are available from the corresponding author upon reasonable request.
