# Supplementary material for: Psidium Defenses Against Meloidogyne enterolobii: Proteomic and Microscopic Analysis of this Plant‐Predator Association
Source: Proteomics. 2025 Jul 26;25(16):17–27. doi: 10.1002/pmic.70015 (PMC12381910; doi:10.1002/pmic.70015)
Supplement: Supplementary file 1 — Supporting Information file 1: pmic70015‐sup‐0001‐SuppMat.docx [file PMIC-25--s001.docx]

**Supplementary Material S1**

**(Costa et al.)**

**Mass Spectrometry Procedures and Conditions**

The runs consisted of three biological replicates of 2.0 µg of peptides. Samples were loaded onto a nanoAcquity UPLC M-Class Symmetry C18 trap column (5 µm, 100 Å, 180 μm × 20 mm, 2D) at a flow rate of 5 µL/min for 3 min, then onto a nanoAcquity M-Class HSS T3 analytical column (1.8 μm, 100 Å, 75 μm × 150 mm) at 400 nL/min and at 45°C.

Peptides were eluted by a varying binary gradient between a mobile phase A (water with 0.1% formic acid), and a phase B (acetonitrile with 0.1% formic acid) as follows: 5% B for 3 min, increasing from 5 to 41% B over 92 min, increasing from 41 to 97% B over 96 min, holding at 97% B for 100 min, and decreasing to 5% B at 102 min.

Mass spectrometry was performed in positive mode and resolution mode (V mode), with 35,000 full widths at half maximum (FWHM) resolutions and ion mobility, and in data-independent acquisition (DIA) mode. Ion mobility separation (IMS) utilized an IMS wave velocity of 800 m/s (HDMSE); transfer collision energy increased from 19 to 55 V in high-energy mode; cone and capillary voltages were 30 V and 3000 V, respectively; and the source temperature was 100°C. For time-of-flight (TOF) parameters, scan time was set to 0.5 s in continuous mode, and the mass range was from 50 to 2000 Da. Human fibrinopeptide B [Glu1] at 100 fmol µL^-1^ was used as an external calibrant, and lock mass acquisition was performed every 30 s. Mass spectrum was acquired using MassLynx software (version 4.1, Waters).

**Proteomic data analysis**

The HDMSE analysis are followed these parameters: 150 counts for low-energy threshold; 50 counts for elevated-energy threshold; 750 counts for intensity threshold; one missed cleavage; minimum fragment ions per peptide equal to three; minimum fragment ions per protein equal to seven; minimum peptides per protein equal to two; fixed carbamidomethyl (C) modifications and variable oxidation (M) and phosphorylation (STY) modifications; false discovery rate (FDR) set to 1%; automatic peptide and fragment tolerance.

Parameters for label-free quantification analysis were: peptide and protein FDR 1%; minimum peptide sequence length of at least six amino acid residues; and minimum peptide score equal to six. The samples were treated by a multidimensional normalization process and the software performed relative protein quantification based on the TOP3 method. Based on the relative abundances of uniquely assigned peptides, the abundances of shared peptides were redistributed to their respective originating proteins, followed by TOP3-based quantification [20].

**Fig. 1S** Images obtained by transmission electron microscopy of the developmental phases of the nematode *Meloidogyne enterolobii* present in the inoculum used in the experiment of this study: eggs (A, B and C); eggshell (D); a juvenile individual emphasizing its anterior feeding region (E) and its posterior region (F).

**Fig. 2S** Volcano plot displaying the distribution of 26 proteins in *P. guajava* and 11 in *P. guineense* between the time points of 5 and 20 DAI: (A) e (B).
